# INJURIES DURING THE COVID-19 PANDEMIC IN THE 2021 PAULISTA SOCCER CHAMPIONSHIP IN BRAZIL

**DOI:** 10.1590/1413-785220243206e279169

**Published:** 2025-01-10

**Authors:** Gustavo Gonçalves Arliani, Danilo José Leite Gomes, Paulo Henrique Schmidt Lara, Jorge Roberto Pagura, Moisés Cohen

**Affiliations:** 1. Escola Paulista de Medicina, Centro de Traumatologia do Esporte, Sao Paulo, SP, Brazil.; 2. Escola Paulista de Medicina, Departamento de Ortopedia e Traumatologia, Sao Paulo, SP, Brazil.; 3. Faculdade de Medicina do ABC, Departamento de Neurologia e Neurocirurgia, Santo Andre, SP, Brazil.

**Keywords:** Soccer, Athletes, Injuries, Epidemiology, Futebol, Atletas, Lesões, Epidemiologia

## Abstract

Objective: To assess the incidence and characteristics of injuries that occurred during the 2021 season of the Paulista Soccer Championship during the coronavirus disease 2019 pandemic and to compare these characteristics before and after the championship interruption. Methods: A prospective study was conducted using an electronic form developed by the Medical Committee of the Paulista Soccer Federation. The results were sent by the team physicians of Series A1 after each round of the Paulista Soccer Championship. Results: Series A1 presented 7.2 injuries per 1,000h of game time. Most injuries occurred within 31–45 min of the match, with muscle injuries being the most frequent and the lower limbs the most affected. Only 10% of injuries required surgery. The strikers were the most affected players and most injuries occurred in penalty-free movements. There was no statistical difference between pre- and post-interruption of the championship due to the pandemic. Conclusion: The incidence of injuries per 1,000h was below the average reported in the literature. Most injuries occurred in the lower limbs; muscle sprains were the most common type of injury, followed by sprains and fractures. MRI was the most commonly requested examination; and most injuries were classified as moderate. Overall, 10% of the injuries were treated surgically. There was no difference between pre- and post-championship interruption. *Level of evidence VI, Descriptive epidemiology study.*

## RESUMO

Objetivo: Avaliar a incidência e características das lesões ocorridas durante a temporada de 2021 do Campeonato Paulista de Futebol que ocorreu durante a pandemia do Covid-19 e comparar as características antes e depois da pandemia dessa doença. Metodologia: Estudo prospectivo que utilizou um questionário eletrônico desenvolvido pelo Comitê Médico da Federação Paulista de Futebol. Os resultados foram enviados pelos médicos das equipes da Série A1 após cada rodada do Campeonato Paulista de Futebol. Resultados: Série A1 apresentou 7.2 lesões por 1000 horas de tempo de jogo. Maioria das lesões ocorreu entre 31-45 minutos de jogo, com lesões musculares sendo as mais comuns e o membro inferior o mais acometido. Apenas 10% das lesões necessitaram de cirurgia. Os atacantes foram os jogadores mais comumente lesionados, e a maioria das lesões ocorreu em situações em que não foi assinalada infração. Não houve diferença estatística em relação às lesões nos períodos antes e após interrupção pela pandemia do Covid-19. Conclusão: A incidência de lesões por 1000 horas de tempo de jogo foi abaixo da reportada na literatura. Maioria das lesões ocorreu nos membros inferiores; lesões musculares foram o tipo mais comum de lesão, seguido de entorses e fraturas. Ressonância foi o exame de imagem mais comumente solicitado, e a maior parte das lesões foi classificada como moderada. Não houve diferença entre antes e após interrupção pela pandemia nas características das lesões. *Nível de evidência VI, Estudo epidemiológico descritivo.*

## INTRODUCTION

 Among different sports, soccer has the largest number of practitioners worldwide, with approximately 200,000 professional athletes and at least 240 million amateur athletes covering all age groups of both sexes. ^1^ This sport demands quick acceleration and deceleration, changes in direction, jumping, and increased physical contact. Due to these characteristics, soccer presents with, in absolute terms, a relatively high number of injuries and thus arouses corresponding interest in sports traumatology. ^2^ ^,^ ^3^

 High-performance sports have undergone significant changes, such as increased physical demand and the subsequent risk of injury. Epidemiological studies have estimated injury incidence rates of 16–28 and 2–11 on match and practice injuries, respectively, for every 1,000h of exposure at the professional level. ^4^ ^,^ ^5^ In soccer, for example, it has been estimated that the injury-related absences of athletes of the major European soccer leagues result in the loss of approximately 500,000€ per month to teams, in addition to compromising the team’s performance and success during a championship season. ^6^

 Previous studies reported that muscle injuries, contusions, contractures, and sprains represent approximately 75% of injuries in professional soccer players, with most affecting the lower limbs, especially the thighs, knees, and ankles. ^6^ In addition, contusion is the most common type of traumatic knee injury. The lateral ankle sprain is also one of the most common joint injuries in soccer and exposes high rates of persistent symptoms and relapses, with consequent local morbidity and a decrease in the athlete’s sports performance. In the thighs, the hamstring muscle strain is the most prevalent injury in soccer, representing 12% of all injuries in high-level players. ^6^ Other studies have shown that the susceptibility to certain types of injuries varies according to the position of each athlete in a match, possibly due to inherent changes in game style and intensity, as the roles involved in each position require specific technical, physiological, and tactical demands. ^7^

Thus, it is presumed that the incidence of this type of injury is higher in male soccer athletes, principally during matches, thus having more risk exposure to muscle injuries, especially in the lower limbs.

## METHODS

This study was approved by the Research Ethics Committee of our institution (number: 1,660,701). This prospective study applied an electronic form, developed by the Medical Commission of the Paulista Soccer Federation (FPF). Patient data were anonymously extracted, and the results were sent by the physicians to the A1 series teams after each round of the 2021 Paulista Soccer Championship.

 This form was developed to analyze the incidence and characteristics of soccer-related injuries and it contained 15 questions about the specifics of the match, athlete, and injury (Appendix 1). The definition used to determine a soccer injury was based on the statement by Fuller et al. for the 2005 FIFA consensus, as follows: “Any physical complaint sustained by a player that results from a football [soccer] match or football [soccer] training, irrespective of the need for medical attention or time loss from football [soccer] activities”. ^8^

The research subjects were players from 16 clubs participating in the 120th edition of the main division of the Paulista Soccer Championship, which is this sport professional competition in the state of São Paulo, that occurred from February 27th to May 23rd, 2021, organized by the FPF. The schedule of each match was registered by the FPF, which is the oldest soccer league in Brazil, being held uninterruptedly since 1902. Forms were completed by the physicians from each team after the matches as of their return from the field and were used to analyze the outcome of each reported injury. Eight questions were structured in the form of complementary examinations and final diagnoses (Appendix 2). The time of each match was registered by FPF (morning: departures starting before 12 p.m., afternoon: departures before 6 p.m., and night: departures after 6 p.m.). After the first three rounds of the A1 series, Paulista Soccer Federation (FPF) stopped the championship for 15 days due to the worsening coronavirus disease 2019 (COVID-19) infection rates in Brazil. The remaining matches, including the finals, were played afterwards.

**Figure 1.**
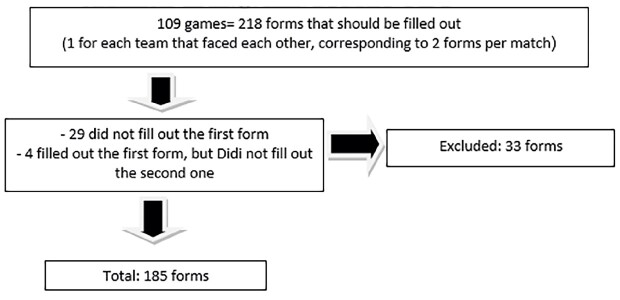
Adressing inclusion and exclusion criteria.

 To obtain data, a form was initially sent to inquire whether there was an injury. In the cases in which the physician reported an injury, another form was sent inquiring the injury details: if any imaging examination was performed, how long the athlete was kept away from training, and if surgical intervention was necessary. Cases of physicians not answering the first form or the second form even though they reported an injury in the first one were excluded ( Figure 1 ). 

 The incidence of injuries was calculated to assess the risk, expressed as the number of injuries per 1,000h of exposure. ^8^ ^,^ ^9^ The following formula was used to calculate the exposure:

$$Exposure\;=\;\frac{no.\;of\;matches\;*\;no.\;players\left(22\right)\;*\;match\;duration\;in\;minutes\;\left(90\right)}{60}$$

Whereas the formula below was used to calculate the incidence at matches:

$$Incidence=\frac{no.\;of\;lesions\;in\;matches}{time\;of\;exposure}*1000$$

### Statistical analysis

Parametric statistics were used for quantitative and continuous data. Two-tailed tests were used to characterize the relative frequency distribution of qualitative variables. Differences were considered statistically significant at p<0.05. Statistical Package for the Social Sciences version 20.0 (IBM Corp., Armonk, NY, USA), Minitab (Minitab, LLC, State College, PA, USA), and Excel Office 2010 (Microsoft Office 2010, Microsoft Corporation, Redmond, WA) were used to perform the analyses.

## RESULTS

### Injury characteristics

Overall, there were 109 games in the championship. Therefore, 218 forms should have been completed (1 for each team that faced each other, corresponding to 2 forms per game, one for each team’s responsible physician). However, of these 218 forms, 29 first forms (13.3%) were not filled out and 4 second forms (1.7%) were not filled out despite the injury report in the first form; thus, 185 (85%) forms were completely filled.

**Figure 2.**
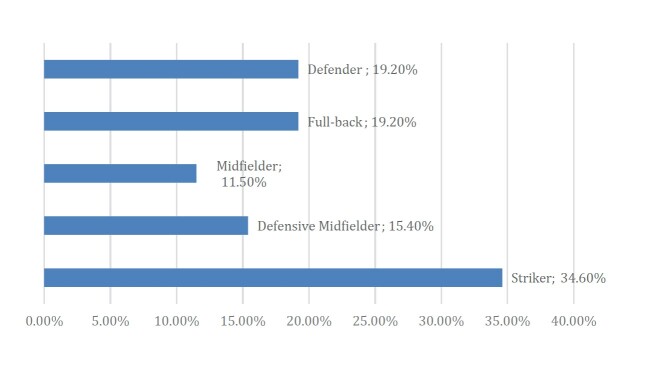
Player positions.

 The average age of injured players was 24.6 years, and the average time of absence due to injury was 20.2 days. Most matches occurred at night (77.1%), while 20.2% and 2.8% occurred in the afternoon and morning, respectively. A total of 26 injuries were reported during all 109 matches, corresponding to an average of 0.24 injuries per match. Most injuries occurred on penalty-free movements (81.8%). In terms of game position, 34.6% of the injuries were experienced by strikers, 19.2% by defenders, 19.2% by full-backs, 15.4% by wide midfielders, and 11.5% by central midfielders, with no injuries to goalkeepers ( Figure 2 ). 

**Figure 3.**
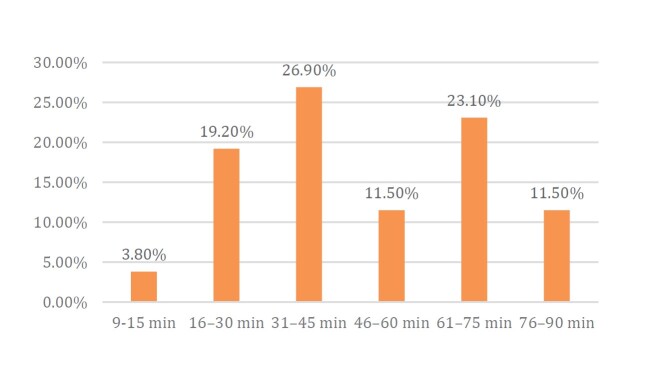
Time interval of injury occurrence

 Most injuries occurred within the 31–45min time interval (26.9%), followed by 61–75min (23.1%) and 16–30min (19.2%) ( Figure 3 ). 

Regarding the distance of the matches, most injuries occurred during home games (46.2%), followed by distances of up to 200km (30.8%); 200–400km (15.4%); and >400km for the matches (7.7%). Injuries occurred mostly in matches played at night with clear weather (69.2%), followed by injuries occurring in rainy weather (11.5%). The mean temperature during the match was lower in the injured group (22.9 °C) than in the uninjured group (23.9 °C), but there were no statistical differences between the injury groups and the analyzed variables.

**Figure 4.**
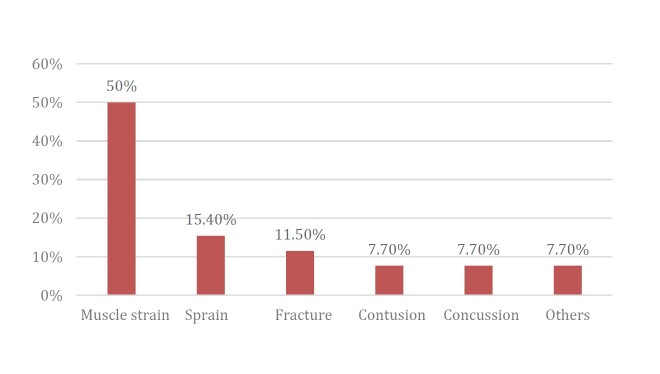
Tipe of injury

 A rate of 7.2 injuries per 1,000 h of matches in Series A1 was observed in 2021. The most common injury sites were the thigh (42.3%), ankle (19.2%), and head/face (11.5 %). Injuries occurred more frequently on the dominant side (53.8%). The most common type of injury was muscle strain (50%), followed by sprains (15.4%), and then fractures (11.5%), contusion (7.7%), concussion (7.7%), and others characterized by dislocation (3.8%) and laceration ( Figure 3 ). Regarding the final diagnosis, the most frequent injuries were hamstring muscle strain (40%), quadriceps muscle strain (10%), and ankle sprain (10%) ( Figure 4 ). 

**Figure 5.**
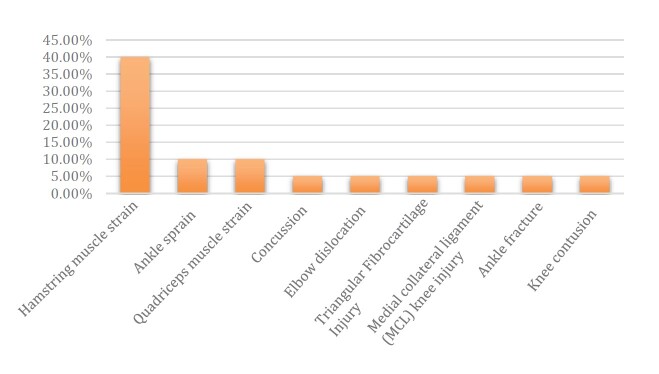
Final diagnoses

### Injury treatments

**Figure 6.**
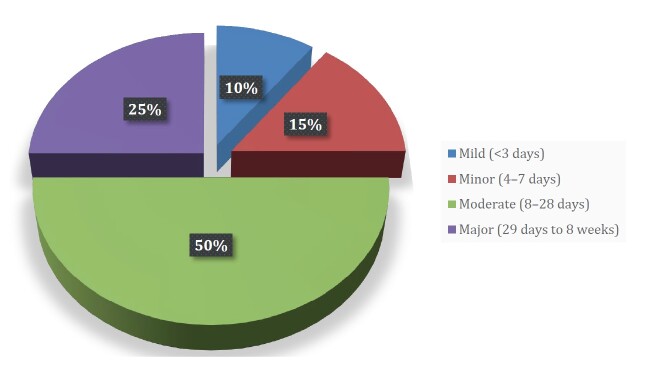
Injury severity.

 When requested, the most frequent complementary examinations were magnetic resonance imaging (MRI) (40%), followed by ultrasonography (35%) and radiography (20%) — no specific protocol was used for the imaging exams. No examination was required for 5% of injuries. Surgery was required for foot and ankle fractures, corresponding to 10% of all registered injuries. Most injuries were considered moderate according to the severity scale, with a medical leave ranging from 8 to 28 days (50%) ( Figure 6 ). 

### Before and after the championship interruption

 The championship was paused from March 15th to March 30th, with 31 (28.4%) and 78 (71.6%) matches played before and after the interruption, respectively, including the finals. To perform this analysis, we used the Chi-square test to compare pre and post championship interruption for injury distribution. The main results are presented in Table 1 . 

**Table 1.** Comparisons of injury distribution before and after championship interruption.

	Before Interruption		After Interruption		Total	
	N	%	N	%	N	%
No Injury	55	88.7%	105	85.0%	160	86.2%
With Injury	7	11.3%	18	15.0%	25	13.8%
Total	62	32.8%	123	67.2%	185	100%

p = 0.492

Note that there were no statistically significant differences in the occurrence of injuries between the two periods, in which a reduction in the rate from 88.7% to 85.0% is noted.

## DISCUSSION

 The study of injuries in professional soccer is important due to their influence on the team final results in national and international tournaments; moreover, understanding the characteristics of these injuries may allow for the implementation of preventive strategies to increase the team’s chances of gaining recognition, such as prizes and titles. ^10^ In several competitions worldwide, epidemiological investigations were performed to identify the patterns of common practices in the main leagues, world tournaments, and World Cups. ^11^ Although soccer is the most popular sport in Brazil, few epidemiological studies have been conducted, and data are scarce on injuries in the main regional and national leagues in the country. ^3^

 In early March 2020, the World Health Organization declared the COVID-19 pandemic, an infection caused by the severe acute respiratory syndrome coronavirus 2. Due to the state of global public health, security protocols were implemented by countries to avoid agglomerations of people to reduce viral transmission. Thus, several soccer championships ^12^ and the collective training of the teams were halted indefinitely. Thus, most athletes had to train at home while following conditioning and strength routines provided by the teams. Despite this, however, many players still displayed signs of detraining, increasing the risk of injuries after returning to the sport, necessitating a multi-component soccer training program including aerobics, endurance, balance, coordination, and power-specific motor skills. ^12^ ^,^ ^13^

This study main findings were an incidence of 7.2 injuries per 1,000 hours of gameplay, with most injuries in lower limbs. The mean number of days the athletes were kept away from practice and matches per injury was 20.2. Most injuries occurred during the last 15 minutes of the first half of the match, and only 10% of the injuries required surgical treatment.

 The incidence of injuries in this study was lower than that reported in the literature, although the characteristics of the injuries were similar. ^3^ ^,^ ^11^ ^,^ ^14^ ^,^ ^15^ ^,^ ^16^ This reduction in incidence may be due to reduced exposure, as well as the preventive measures implemented by the clubs and a better condition of the soccer fields. Muscle strains and sprains accounted for most injuries (65%), which could be associated with the increased demand on the player’s performance, following a general trend in soccer, a result similar to that reported previously. ^17^ ^,^ ^18^ ^,^ ^19^ Most injuries were treated conservatively, with only 10% requiring a surgical approach. 

 Similar to other studies developed by our group, magnetic nuclear resonance imaging was the most requested examination. ^3^ ^,^ ^11^ ^,^ ^16^ As most injuries occurred in muscular (50%) and ligamentous (15%) tissues, thus magnetic resonance imaging is the most useful method for these injuries investigation. Most injuries occurred within the 31–45min time interval of the first half of the match. ^16^ ^,^ ^11^ ^,^ ^3^ In other studies, most injuries occurred in the last 30 min of the final half of the match. ^17^ ^,^ ^3^ However, in some of these studies, the tournaments were organized in a simple elimination system, in which each game defined the team classification or elimination, most likely contributing to the increased athlete endeavor and increased risk of injury in the final minutes of the matches. 

Evaluation of the incidence rates showed injury rates of 11.3% and 15%, respectively, before and after the championship interruption. In both periods, muscle strain was the most common injury, accounting for almost half of the cases (43% before and 52.6% after the interruption), indicating that the interruption did not change the injury characteristics. Furthermore, differences in the injury occurrence were observed. Before the interruption, injuries were more common within the 31–45min time interval of the match (43%). After the championship return, injuries occurred mostly within three different time periods: 16–30min; 31–45min; and 61–75min; in which each period accounted for 21% of the injury occurrence. However, no significant differences were observed in the evaluated factors.

 Pucsok et al. ^14^ showed that home training during the championship interruption effectively improved aerobic fitness but did not allow players to maintain their usual speed endurance. Grazioli et al. ^20^ reported that 63 days of quarantine impaired the various physical performance abilities compared to the normal period between seasons. These findings can be justified by the number of limited locomotor movements, which ends up impairing the speed endurance capacity that is one of the vital components of current soccer performance. Special attention should be paid to body composition, speed, and power-related capabilities after long-term detraining. Moreno-Pérez et al. ^21^ showed decreased eccentric strength of the hamstrings during isolation at home; this magnitude of muscle weakness may indicate an increased risk of injury. Despite the increased risk of injuries, we observed no significant difference between the incidence and type of injuries and the time that they occurred. 

 Recent studies have demonstrated the impact of interruption on the physical qualities of athletes. ^12^ ^,^ ^13^

 In comparison with other popular sports in Brazil, a study by Rosa et al. ^22^ addresses predictors of injuries in Brazilian athletes of other sport categories . Female handball, followed by male volleyball, presented anterior cruciate ligament (ACL) injury as the most prevalent. Isolated ACL injury occurred in 37 (6.3%) of the 585 athletes in the study. The incidence in women was slightly higher compared to that in men, with 18 cases (7.2%) in 249 athletes. Regarding men, there were 19 injuries (5.6%) in 336 athletes. 

This study limitation was the reliability of the information provided by the clubs’ medical teams, as well as the lack of official records of injuries occurring during matches. Furthermore, it was not possible to accurately measure the exposure of each athlete.

## CONCLUSION

The incidence of injuries per 1,000 game hour was below the average reported in the literature. Most injuries occurred in the lower limbs. Muscle strains were the most common type of injury, followed by sprains and fractures. Magnetic resonance imaging was the most commonly requested examination. Most injuries were classified as moderate. Ten percent of the injuries were treated surgically. The results were similar before and after the championship interruption due to the COVID-19 pandemic. This study findings indicate possible preventive measures to reduce or minimize the number and severity of soccer injuries since it addresses probable risk factors.

## APPENDIX 1

Mapping of the Injuries in the 2021 Paulista Soccer Championship

REPORT ON ORTHOPEDIC INJURIES OCCURED DURING THE 2021 PAULISTA SOCCER CHAMPIONSHIP

1) THIS REPORT REFERS TO THE FOLLOWING MATCH:

_________________________________________________________________________________________________________________________________________

2) WHAT WAS THE WEATHER LIKE AT THE TIME OF THE MATCH?

( ) SUNNY

( ) CLOUDY

( ) RAINY

( ) SUN SHOWER

( ) RAIN AND LIGHTNING

( ) NIGHT – CLEAR SKY

( ) NIGHT –RAINY

3) TEMPERATURE MEASURED AT THE TIME OF THE MATCH:

_________________________________________________________________________________________________________________________________________

4) LOCATION

( ) HOME GAME

( ) UP TO 200KM AWAY

( ) FROM 200 TO 400KM AWAY

( ) MORE THAN 400KM AWAY

5) WERE THERE INJURIES SUSTAINED DURING THE MATCH?

( ) YES

( ) NO

**FILL OUT THE FOLLOWING ITEMS IN CASE OF REPORTED INJURIES**

6) NAME OF THE INJURED ATHLETE:

DATE OF BIRTH_______________________________________________________________________________________________________________________________

7) ATHLETE’S POSITION:

( ) GOALKEEPER

( ) CENTRAL DEFENDER

( ) FULL-BACK

( ) WIDE MIDFIELDER

( ) CENTRAL MIDFIELDER

( ) STRIKER

8) WHEN WAS THE INJURY SUSTAINED?

( ) 0–15 MIN

( ) 15–30 MIN

( ) 30–45 MIN

( ) 45–60 MIN

( ) 60–75 MIN

( ) 75–90 MIN

( ) OVERTIME – 1 ^ST^ HALF

( ) OVERTIME – 2 ^ND^ HALF

9) DID THE INJURY OCCUR AFTER CONTACT OR COLLISION WITH THE BALL, GOAL, OR WITH ANOTHER ATHLETE?

( ) YES

( ) NO

10) IF YES, IN WHICH CIRCUMSTANCES?

( ) ANOTHER PLAYER

( ) BALL

( ) GOAL

( ) OTHERS

11) DID THE REFEREE CONSIDER THE INJURY MECHANISM A MISCONDUCT?

( ) YES

( ) NO

12) WHAT PUNISHMENT WAS APPLIED?

( ) FOUL, NO CARD.

( ) FOUL AND YELLOW CARD

( ) FOUL AND RED CARD

( ) NO PENALTY

13) WHERE WAS THE INJURY SUSTAINED?

( ) HEAD

( ) TRUNK

( ) UPPER LIMBS/EXTREMITIES

( ) LOWER LIMBS/EXTREMITIES

( ) N/A

14) SIDE OD THE INJURY

( ) RIGHT

( ) LEFT

( ) N/A

( ) BILATERAL

15) TYPE OF INJURY

( ) Strain

( ) Sprain

( ) Contusion

( ) Fracture

( ) Joint Dislocation

( ) Wound (with contusion)

( ) Concussion

( ) Cramp

( ) Others

PROBABLE FINAL DIAGNOSIS:

_________________________________________________________________________________________________________________________________________

## APPENDIX 2

Injury Report: 2021 Paulista Soccer Championship

REPORT ON ORTHOPEDIC INJURIES SUSTAINED DURING THE 2021 PAULISTA SOCCER CHAMPIONSHIP

1) NAME OF THE INJURED ATHLETE

DATE OF BIRTH:___________________________________________________________________________________________________________________

POSITION:_________________________________________________________________________________________________________________________

INJURY:____________________________________________________________________________________________________________________________

DATE OF THE INJURY:___________________________________________________________________________________________________________________

2)COMPLEMENTARY TESTS/EXAMS REQUESTED:

( ) NONE

( ) RADIOGRAPHY (RX)

( ) ULTRASOUND (US)

( ) CAT SCAN

( ) MRI

( ) S:___________________________________________________________________________________________________________________

3) DID THE INJURY REQUIRE SURGERY?

( ) YES

( ) NO

4) IF YES, SPECIFY:

_____________________________________________________________________________________________________________________________________

5) ATHLETE’S RETURN DATE TO SPORTS ACTIVITIES:

_____________________________________________________________________________________________________________________________________

6) DAYS KEPT AWAY FROM PRATICE:

_____________________________________________________________________________________________________________________________________

7) INJURY SEVERITY SCALE:

( ) MILD (UP TO 3 DAYS AWAY)

( ) MINOR (3 TO 7 DAYS AWAY)

( ) MODERATE (7 TO 28 DAYS AWAY)

( ) MAJOR (7 DAYS TO 8 WEEKS AWAY)

( ) SEVERE (MORE THAN 8 WEEKS OF TIME LOSS)

8) DID THE FINAL DIAGNOSIS CONFIRM THE INITIAL DIAGNOSIS?

( ) YES

( ) NO

FINAL DIAGNOSIS:

_____________________________________________________________________________________________________________________________________
